# Postmarketing Safety Surveillance and Reevaluation of Danhong Injection: Clinical Study of 30888 Cases

**DOI:** 10.1155/2015/610846

**Published:** 2015-10-05

**Authors:** Xue-Lin Li, Jin-Fa Tang, Wei-Xia Li, Chun-Xiao Li, Tao Zhao, Bu-Chang Zhao, Yong Wang, Hui Zhang, Xiao-Fei Chen, Tao Xu, Ming-Jun Zhu

**Affiliations:** ^1^The First Affiliated Hospital of Henan University of Traditional Chinese Medicine, Zhengzhou 450000, China; ^2^Shandong Buchang Pharmaceutical Co., Ltd., Heze 274000, China

## Abstract

Traditional Chinese medicine injections (TCMIs) have played an irreplaceable role for treating some clinical emergency, severe illness, and infectious diseases in China. In recent years, the incidence rates of adverse drug reactions (ADRs) of TCMIs have increased year by year. Danhong injection (DHI) is one representative TCMI comprised of Danshen and Honghua for treating cardiovascular and cerebrovascular diseases in clinic. In present study, the postmarketing safety surveillance and reevaluation of DHI were reported. Total 30888 patients in 37 hospitals from 6 provinces participated in the study. The results showed that the ADR incidence rate of DHI was 3.50‰. Seventeen kinds of new adverse reactions of DHI were found. The main type of ADRs of DHI was type A (including sweating, dizziness, headache, flushing, vasodilation, eye hemorrhage, faintness, chest pain, palpitations, breathlessness, anxious, nausea, flatulence, vomiting, hypotension, hypertension, local numbness, dyspnea, joint disease, and tinnitus) accounting for 57.75%. The severities of most ADRs of DHI were mild and moderate reactions accounting for 25.93% and 66.67%, respectively. The main disposition of ADRs of DHI was drug withdrawal and without any treatments. The results can provide basis for amendment and improvement of the instructions of DHI, as well as demonstration and reference for the postmarketing safety surveillance and reevaluation of other TCMIs. And the rationality, scientificity, and safety of clinical applications of TCMIs could be improved.

## 1. Introduction

Traditional Chinese medicine injection (TCMI) is prepared by extracting and purifying effective substances from herbs (or decoction pieces) with modern scientific techniques and methods. Compared with orally administrated herb medicines, the injection is a new form of TCM preparations including solutions, emulsions, powder, or concentrated solutions [1, 2]. It has played an irreplaceable role in the treatment of some clinical emergency, severe illness, and infectious diseases [3–6].

In recent years, the incidence rates of adverse drug reactions (ADRs)/adverse drug events (ADEs) of TCMs have increased gradually along with the wider application and increasing varieties of TCMs. It is worth noticing that ADRs/ADEs caused by TCMIs are particularly prominent [7]. ADRs/ADEs are a worldwide problem and are one of the leading causes of mortality and morbidity in health care facilities worldwide [8, 9]. They can significantly impede a patient's adherence to treatment and in turn diminish the therapeutic benefit, potentially reducing health and quality of life. Therefore, understanding ADRs/ADEs of TCMIs is essential for managing unintended outcomes and achieving successful treatment. When one kind of TCMIs comes into the market, its safety profile is always little known. Thus, it is very necessary to carry out the postmarketing safety surveillance and reevaluation of TCMIs.

A representative injection is Danhong injection (DHI), which was awarded the first Chinese medicine patent gold medal in the year of 2010. DHI showed 3 consecutive annual sales of more than 1 billion yuan RMB, reaching 3 billion in 2011, and had become the top Chinese medicine for cardiovascular and cerebrovascular diseases in China [10]. DHI is a standardized water-soluble product manufactured from the root and rhizome of* Salvia miltiorrhiza* Bge. (Danshen, officially recognized in the Chinese Pharmacopoeia as Salviae Miltiorrhizae Radix et Rhizoma) and the flower of* Carthamus tinctorius* L. (Honghua, officially recognized in the Chinese Pharmacopoeia as Carthami Flos). It is a famous Chinese medicinal formula which is used extensively for treating cardiovascular and cerebrovascular diseases in clinic (such as coronary heart disease, angina, myocardial infarction, ischemic encephalopathy, and cerebral thrombosis) due to its traditional Chinese medical effects of activating blood circulation, dissipating blood stasis, and dredging meridians and collaterals [11].

However, the postmarketing safety of DHI is relatively little known [12–14]. In our previous studies, four assessment methods of postmarketing safety on DHI were compared, and the results showed that centralized hospital monitoring was an appropriate method to carry out postmarketing safety evaluation of TCMIs [15]. Therefore, the postmarketing safety (including the incidence rate, types, severities, and other information of ADRs/ADEs) of DHI with 30888 cases was further investigated by a trained physician and pharmacist team by using the centralized hospital monitoring method, which could objectively reflect the real world of clinical applications.

## 2. Subjects and Methods

### 2.1. Ethical Approval

The experimental protocol was reviewed and approved by the Ethical Committee of The First Affiliated Hospital of Henan University of TCM and was conducted according to the principles expressed in the Declaration of Helsinki.

### 2.2. Subjects

Total 30888 patients administrated of  DHI from 37 hospitals in 6 provinces participated in the study between April 1, 2009, and August 30, 2013.

### 2.3. Drug

DHI was manufactured by Shandong Buchang Pharmaceutical Co., Ltd. (Heze, Shandong, China), which was one of the exclusive varieties.

### 2.4. Methods

#### 2.4.1. The Training of Safety Monitoring

Centralized hospital monitoring method was used to reevaluate the postmarketing safety of DHI. Pharmacists as a third party who did not interfere with the normal clinical treatment of doctors went to ward every day to carry out the safety monitoring of each in-patient administered DHI during the therapeutic period. At the beginning of research, all clinical pharmacists who participated in the study must accept the unified training of safety monitoring. The training of safety monitoring was organized by Shandong Buchang Pharmaceutical Co., Ltd. and was carried out by total research group (The First Affiliated Hospital of Henan University of TCM and Shandong Buchang Pharmaceutical Co., Ltd.). Director of pharmacy, quality control personnel, and monitoring staff of each monitoring hospital all should participate in the training. Most of the quality control personnel and monitoring staff were clinical pharmacists; some of them were pharmacists-in-charge. The training content included monitoring process and filling requirements of “Monitoring Information Form.” After training, all trainers should participate in the examination, and the trainers whose scores are less than 80 points should participate in the training again or cancel their participation. All examination papers were saved by the total research group.

#### 2.4.2. The Filling Requirements of “Monitoring Information Form”

The filling requirements of “Monitoring Information Form” mainly included the following. (1) Please use black ink or ball-point pen to fill out the “Monitoring Information Form”. In order to prevent Carbonless printing to the next page, please put the pads on top of that page which will be filled out. (2) Items in the “Monitoring Information Form” should be carefully and truthfully filled out. Those contents should be filled in or written neatly and clearly, which also should be legible and accurate. If an error occurred, please draw a single line above the errors, write the correct answer above or next to those errors, and write the modifier's name and modified date on the upper right corner of the correct answer. (3) All items should be filled out. Choice questions that were not marked as multiple choice were all single choice. Please fill the correct answer code in the □. And all horizontal lines __________ should be answered with words. (4) The items which were “not done” should be filled with “ND”; “do not know” should be filled with “UK.” (5) The “start time of drug administration” should be accurate to minute. (6) Names of all drugs should be filled with their generic names, rather than their trade names. The dosage form of drugs should be shown in parentheses following the name of drugs. Dosage unites should be written clearly, such as “mg, mL, tablets, pills.” (7) The “Monitoring Information Form” was in duplicate. The first form associated with cover should be retained by the manufacturer. The second copy form should be retained by each monitoring hospital.

The basic information (including name, gender, age, nationality, body weight, bad habits, drug allergy and whether it was the first time of administration of DHI), syndrome, dose regimen, adverse reactions, efficacy, laboratory testing, and rationality evaluation of drug administration of 30888 patients were mainly observed and recorded. Then, all information was entered into the HIS (hospital information system) database, which was used for the further statistical analysis. All analyses were performed by using the SPSS statistical software package (version 16.0).

#### 2.4.3. The Process of Postmarketing Safety Reevaluation of DHI

**Figure 1:**
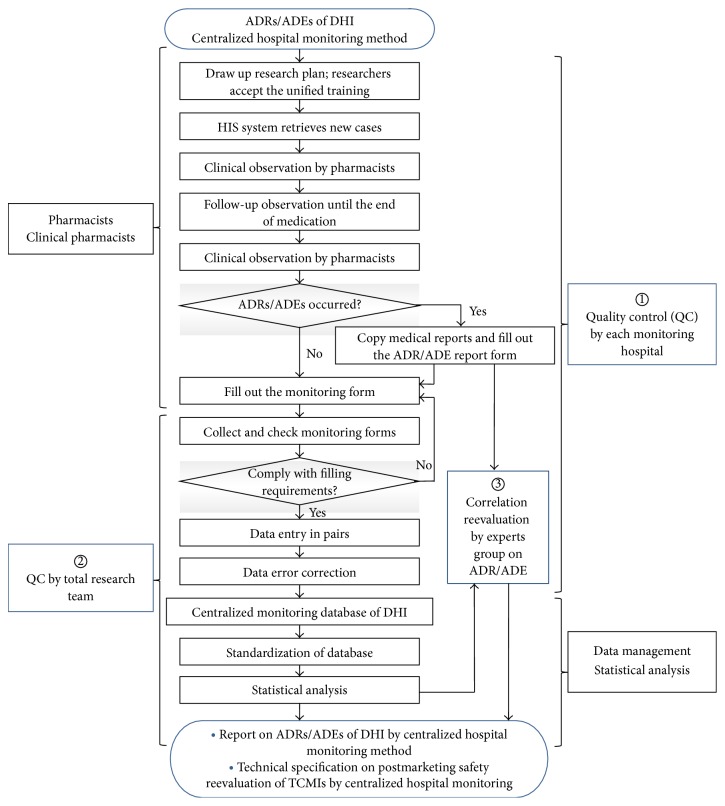
The overall experimental flow chart of postmarketing safety surveillance and reevaluation of DHI.

In order to guarantee the objectivity and accuracy of ADR results, 3 grades evaluation of ADRs and ADEs were conducted in the present study. Primary evaluation: ADRs and ADEs were preliminarily determined by the evaluation team (including the director of pharmacy, quality control personnel, and monitoring staffs) of each monitoring hospital, respectively. Intermediate evaluation: ADRs and ADEs determined by each monitoring hospital were reevaluated by ADRs/ADEs experts of total research team (The First Affiliated Hospital of Henan University of TCM and Shandong Buchang Pharmaceutical Co., Ltd.). Ultimate evaluation: all ADRs/ADEs were evaluated by authoritative clinical and pharmaceutical experts (Chief Pharmacists, Chief Physicians, Deputy Chief Pharmacists, and Deputy Chief Physicians). Final results were comprehensively given by combining with the results of the primary and intermediate evaluations. The overall experimental flow chart is shown in Figure 1.

#### 2.4.4. The Correlation Evaluation between ADRs and ADEs

**Figure 2:**
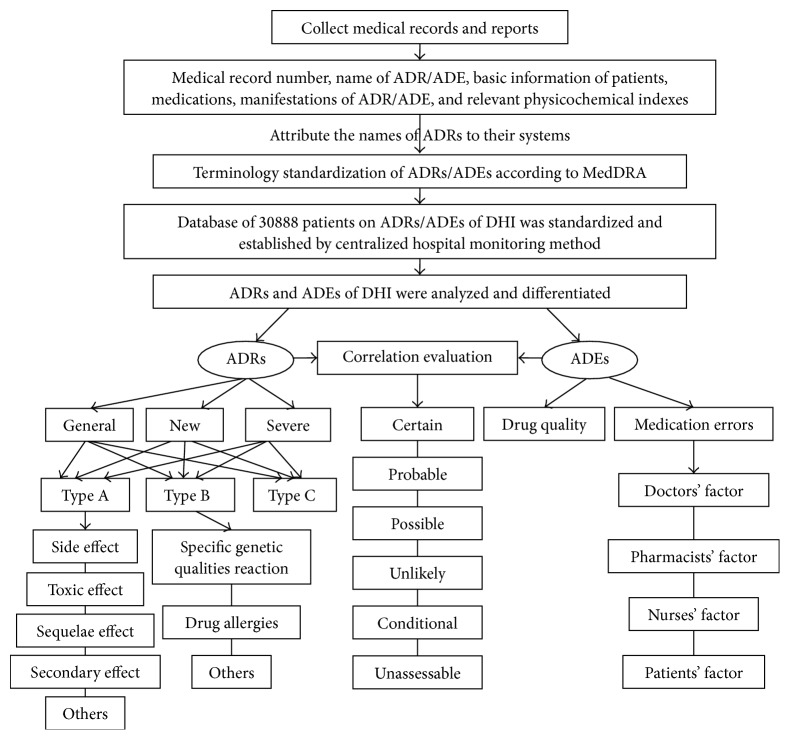
The detail flow chart of correlation evaluation between ADRs and ADEs.

During the process of postmarketing safety reevaluation of DHI, the correlation evaluation between ADRs and ADEs was the focal point. All ADRs/ADEs should be preliminarily differentiated on basis of their definitions, respectively. An ADR is a response to a drug which is noxious and unintended and which occurs at dose normally used in man for prophylaxis, diagnosis, or therapy of disease, or for the modification of physiologic function. ADRs may occur following a single dose or prolonged administration of a drug or result from the combination of two or more drugs. It does not include the reactions caused by accidental or intentional drug overdoses and improper medications. The meaning of ADR differs from the meaning of “side effect,” as the last expression might also imply that the effects can be beneficial. The study of ADRs is the concern of the field known as pharmacovigilance. An ADE refers to any injury occurring at the time of drug administration, whether or not it is identified as a cause of the injury. An ADR is a special type of ADE in which a causative relationship can be shown. The causal relationship with the administration of the investigational drug or a study procedure was assessed according to the categories as described by the Uppsala Monitoring Centre and recommended by the World Health Organization (WHO) (certain, probable, possible, unlikely, conditional, and unassessable) [16–18]. The detail flow of correlation evaluation between ADRs and ADEs is shown in Figure 2.

The explanations of the terms were as follows. (1) Certain: the sequence between medication and ADRs' occurrence was reasonable. ADRs could stop or quickly reduce or turn better after drug withdrawal. ADRs could occur again or increase significantly when drug was readministered. It could be supported by literatures. The primary disease and other factors had been ruled out. (2) Probable: there was no history of repeat medication; others were same as “Certain.” If the investigational drug was administrated by combination with other drugs, the probability of ADR occurrence produced by the combination drugs could be excluded. (3) Possible: there was close relationship between medication and ADEs' occurrence. It could be supported by literatures. But more than one drug could cause the ADRs/ADEs, or the factors of primary disease could not be ruled out. (4) Unlikely: there was no close relationship between medication and ADEs' occurrence. The performances of reactions did not match the known ADRs/ADEs of the investigational drug. The reactions during the development of primary disease might have similar clinical manifestations. (5) Conditional: the contents of “Monitoring Information Form” were not complete, which could be evaluated after the supplementary specification. It was difficult to determine the relationship between cause and effect, which was scant in documentation. (6) Unassessable: many items in the “Monitoring Information Form” were missed. It was difficult to determine the relationship between cause and effect. And the missing items could not be supplemented.

## 3. Results

### 3.1. Primary Evaluation of ADRs of DHI in Each Monitoring Hospital

When the monitoring hospitals were selected, the monitoring hospitals should include general hospitals and Chinese medicinal hospitals. The proportion of general hospitals in all hospitals should be greater than 0.5. Of the 37 monitoring hospitals, there were 31 general hospitals and 6 Chinese medicinal hospitals in the postmarketing safety surveillance and reevaluation of DHI. As shown in Table 1, there were total 132 ADE cases in 30888 patients.

### 3.2. Intermediate Evaluation of ADRs/ADEs

Among the 132 ADE cases, 115 cases (the cases of grades I, II, and III) were determined as the ADRs/ADEs of DHI (Table 2).

### 3.3. Ultimate Evaluation of ADRs/ADEs

Among the 115 ADR/ADE cases of DHI, 108 cases were identified as ADRs of DHI, and 7 cases were identified as ADEs of DHI. Of the 7 ADEs, 2 cases were caused by nurses because of the DHI injection speed; 5 cases were caused by doctors including the improper medications (2 cases), solvents (2 cases), and compatibility (1 cases).

### 3.4. Incidence Rate and Manifestations of ADR

The ADR incidence rate of DHI was 3.50‰. ADRs of DHI involved the damage of several systems. Among them, the damages of skin and its appendages, central and peripheral nervous system, and extracardiac vascular system were more common, which totally accounted for 68.35% (Table 3). There were 17 kinds of new adverse reactions of DHI which were found in the present study, manifesting as sweating, clammy skin, superficial phlebitis, allergic purpura, vasodilation, eye hemorrhage, periorbital edema, faintness, chest pain, anxious, flatulence, cyanosis, hypotension, hypertension, local numbness, joint disease, and tinnitus.

### 3.5. Classification of ADRs

On basis of pathogenesis of ADRs, ADRs are divided into three types (types A, B, and C) by WHO [16, 17]. Type A are predictable adverse reactions which are a consequence of the drug's normal pharmacological effects and dose-related with a low mortality. Such reactions are usually due to incorrect dosage (too much or too long) or disordered pharmacokinetics and failure of drug elimination. Type A usually include side effects, toxic effects, aftermath effects, and sequelae effect. Type B reactions are not predictable from the drug's main pharmacological actions. They are not dose-related and they have a considerable mortality. Type B reactions occur infrequently, which usually include allergies and specific genetic qualities reactions. Type C reactions refer to the abnormal reactions other than types A and B, which usually occur after long-term treatment and have long incubation period. It is difficult to predict type C reactions, which do not have ambiguous relationship with time. The pathogenesis of some type C reactions correlated with carcinogenic, teratogenic, and cardiovascular disease and fibrinolytic system changes after long-term medication.

All adverse reactions in Table 3 could be classified into types A, B, and C. Type A reaction included sweating, dizziness, headache, flushing, vasodilation, eye hemorrhage, faintness, chest pain, palpitations, breathlessness, anxiousness, nausea, flatulence, vomiting, hypotension, hypertension, local numbness, dyspnea, joint disease, and tinnitus. Type B reaction included pruritus, rash, allergic purpura, and periorbital edema. Type C reaction included clammy skin, tics, superficial phlebitis, chills, high fever, fever, and cyanosis.

Several adverse reactions might occur in one patient, which belonged to different types of ADRs. For example, if dizziness and superficial phlebitis occurred in one patient, this patient would have both types A and C, because dizziness belonged to type A, while superficial phlebitis belonged to type C. Among the 108 cases, 55, 31, and 14 cases were classified into types A, B, and C, respectively; 4 cases had both types A and B; and another 4 cases had both types A and C. Therefore, patients with types A, B, and C reactions were 63, 35, and 18 cases, respectively.

### 3.6. Occurrence Time of ADRs

The ADRs of 41, 14, 20, and 33 cases were observed within half an hour, from half an hour to 1 hour, from 1 hour to 24 hours, and over 24 hours, respectively. The shortest of that could be observed in 1 minute after administration of DHI, while the longest of that was found in the 11th day.

### 3.7. Severity Classification of ADRs

The severities of ADRs were divided into three grades, including mild (temporary discomfort and tolerable), moderate (significant discomfort), and severe (potentially life threatening or causing permanent disability or death) reactions [18]. Mild reactions are perceptible symptoms or signs without stopping medication and special treatment, which do not affect the daily life. The symptoms or signs of moderate reactions can be tolerated but need special treatment, which also do not affect the daily life. The symptoms or signs of severe reactions cannot be tolerated, which need to stop medication and special treatment. And the severe reactions can affect the daily life.

Of 108 ADRs, 28, 72, and 8 cases were classified as mild, moderate, and severe reactions, respectively; which accounted for 25.93%, 66.67%, and 7.41%. Among the 8 severe cases, the symptoms of 4 cases had more severe reactions, manifesting as chills, fever, cyanosis, and convulsions.

### 3.8. Disposition of ADRs

Treatment measures against the ADRs of DHI included drug withdrawal, symptomatic treatment for the ADRs, combination method between reducing the dosage of drugs and drug withdrawal, and combination method between drug withdrawal and symptomatic treatment for the ADRs. Among 108 cases, 28 cases recovered without any treatment measures (25.93%); 57, 4, 1, and 18 cases recovered after drug withdrawal, symptomatic treatment, combination method between reducing the dosage of drugs and drug withdrawal, and combination method between drug withdrawal and symptomatic treatment for the ADRs, respectively, which accounted for 52.78%, 3.70%, 0.93%, and 16.67%.

### 3.9. Recovery of ADRs

Among 108 ADRs, 64 cases were completely cured and 46 cases took a turn for the better. There were no sequelae or deaths. Of them, 11, 26, 12, and 59 cases improved within 1 hour (10.19%), 1~6 hours (24.07%), 6~24 hours (11.11%), and over 24 hours (54.63%).

## 4. Discussion

WHO, the Food and Drug Administration (FDA), and the Joint Commission on Accreditation of Healthcare Organizations (JCAHO) have recognized the importance of establishing mechanisms for ADRs/ADEs surveillance in health care organizations [19–21]. Hospitals are mandated to have ongoing drug surveillance programs in place in order to detect and evaluate the effects of drugs and to propagate safe, appropriate, and effective drug therapies [21].

Several methods of surveillance are used in the clinical setting to detect ADRs/ADEs. In our previous study, centralized hospital monitoring method was the appropriate method to carry out postmarketing safety evaluation of TCMIs [15]. This method is one kind of the international advanced research methods of drug epidemiology. It is an observational research method, which can make intensive study of clinic without intervention of clinical applications. This method can timely, comprehensively, and accurately observe the adverse reactions, which is called “the research of real world.” Additionally, the correlation evaluation between ADRs and ADEs could be carried out according to the collected clinical materials, and the accurate incidence rate and types and severity of ADRs/ADEs can be obtained. It is a fast and scientific method of postmarketing safety reevaluation. This method is suitable for monitoring the varieties which have a certain market time and stable amount of applications. Otherwise, the number of cases is too low to achieve the purpose of the evaluation. Due to the restrictions of observation time and funding, it is not easy to find the rare adverse reactions. Therefore, accurate conclusions could be comprehensively obtained by the conjunction with other assessment methods such as the spontaneous reporting method. Voluntary spontaneous reporting systems can be used as a necessary complement of centralized hospital monitoring method. The rare ADRs/ADEs can be found by voluntary spontaneous reporting systems.

There are postmarketing safety surveillance and reevaluations for the western medicine and TCMIs. For example, the postmarketing safety surveillance of Shenmai Injection was reported [22]. The results showed that were 5 ADRs in 699 cases, and the ADR incidence rate of Shenmai injection was 0.72%. Guangdong Pharmacological Society was entrusted with postmarketing intensive monitoring study of Shenqifuzheng injection in 2007 [23]. Their results showed that, among 20100 cases observed, the incidence of ADR was 1.85‰, 27 cases had “mild” ADRs/ADEs, and 10 cases displayed “moderate” ADRs/ADEs. There were no severe ADRs/ADEs. Additionally, the postmarketing safety surveillance data of AS03-adjuvated A (H1N1) pandemic vaccine in Ontario, Canada, was reviewed [24].

Clinical pharmacists play an important role in ADE surveillance activities. Pharmacists' training in therapeutics and comprehensive drug knowledge makes them an obvious choice for ADE surveillance. Pharmacists' knowledge of drugs and clinical therapeutics may give them an advantage over other clinicians for the purpose of inpatient ADRs/ADEs detection. As awareness of patient safety issues increases, pharmacists find themselves more engaged in ADRs/ADEs surveillance activities. It is important to note that, in the studies, pharmacists not only were limited to medication orders or laboratory values but also took into account any textual signals that existed in the medical record, such as progress notes, shift assessments, and pharmacist's notes. Pharmacists play an important role for the rational drug applications and improvement of quality of life of patients.

In our previous study, the ADR incidence rate of 10409 cases of DHI was 6.82‰ [24]. In the present study, the ADR incidence rate of 30888 cases was 3.50‰. The ADR incidence rate of 30888 cases of DHI was lower than that of 10409 cases. Possible reasons were mainly the following aspects: (1) filling and monitoring process of adverse reaction monitoring tables were more standardized; (2) under the guidance of previous monitoring results, the quality of DHI had been improved after the quality control of original ingredients and the optimization of processing technique; (3) the analysis of results was more standardized and accurate; (4) the administration of DHI by doctors and nurses was more standardized and reasonable.

The postmarketing surveillance of TCMIs was a good method to solve information lag of drug instructions. It could supplement the drug instructions, keep up with the latest research progress of related drugs, guide the clinical application of drugs, and improve the safety and effectiveness of drugs in clinical applications.

## 5. Conclusions

The postmarketing safety surveillance and reevaluation of DHI was carried out with 30888 cases from 37 hospitals in 6 provinces. The incidence rate, types, severities, and other information of ADRs/ADEs of DHI were obtained. The research system and mode of postmarketing safety surveillance and reevaluation of TCMIs were established, which can provide demonstration and reference for other TCMIs and improve the rationality, scientificity, and safety of clinical applications of TCMIs.

## Figures and Tables

**Table 1:** The general information and the number of ADE cases in 37 monitoring hospitals.

Hospitals	Number of cases	Constituent ratio (%)	Number of ADE cases	Constituent ratio (%)
The First Affiliated Hospital of Henan University of TCM	9468	30.65	71	53.79
Henan Provincial People's Hospital	600	1.94	3	2.27
The First Affiliated Hospital of Zhengzhou University	3580	11.59	16	12.12
The Second Affiliated Hospital of Zhengzhou University	299	0.97	1	0.76
The Second Affiliated Hospital of Henan University of TCM	1050	3.40	3	2.27
Zhengzhou Municipal People's Hospital	2002	6.48	5	3.79
Shanxi Province Hospital of TCM (Xi'an)	501	1.62	0	0.00
General Hospital of TISCO	199	0.64	0	0.00
Taiyuan City Centre Hospital	398	1.29	2	1.52
Wuhan Union Hospital	942	3.05	0	0.00
Chongqing Third People's Hospital	149	0.48	3	2.27
The Second TCM Hospital of Shanxi Medical College (Taiyuan)	149	0.48	2	1.52
The First Hospital of Shanxi Medical University (Taiyuan)	789	2.55	3	2.27
Chongqing First People's Hospital	239	0.77	4	3.03
Zhongshan Hospital of Chongqing	331	1.07	0	0.00
The Second Affiliated Hospital of Xi'an Medical University	902	2.92	0	0.00
Xi'an 521 Hospital	518	1.68	0	0.00
Xi'an Aerospace General Hospital	506	1.64	0	0.00
Xi'an People's Liberation Army 451 Hospital	999	3.23	2	1.52
Xi'an 141 Hospital	100	0.32	0	0.00
Gaoling County Hospital	299	0.97	0	0.00
Xi'an Central Hospital	500	1.62	4	3.03
Chang'an District Hospital	200	0.65	0	0.00
People's Liberation Army 153 Hospital	1002	3.24	0	0.00
Zhengzhou First People's Hospital	300	0.97	1	0.76
Shanxi Province Hospital of TCM (Taiyuan)	300	0.97	0	0.00
Taiyuan People's Hospital	500	1.62	0	0.00
Shanxi Hospital of Integrated Traditional and Western Medicine (Taiyuan)	420	1.36	1	0.76
The Second Hospital of Hebei Medical University	503	1.63	0	0.00
Shijiazhuang Hospital of TCM	1006	3.26	0	0.00
The First Hospital of Hebei Medical University	503	1.63	0	0.00
Shanxi Provincial People's Hospital (Taiyuan)	300	0.97	2	1.52
People's Liberation Army 323 Hospital	200	0.65	0	0.00
Shanxi Coal Central Hospital (Taiyuan)	496	1.61	0	0.00
Chongqing Cancer Hospital	149	0.48	1	0.76
Wuhan Fifth People's Hospital	100	0.32	1	0.76
Wuhan General Hospital of the Yangtze River Shipping	389	1.26	7	5.30
Total	**30888**	**100.00**	**132**	**100.00**

**Table 2:** Results of correlation evaluation between ADRs and ADEs of DHI.

Grade	Results of correlation evaluation	Number of cases	Constituent ratio (%)
I	Certain	21	15.91
II	Probable	61	46.21
III	Possible	33	25.00
IV	Unlikely	13	9.85
V	Conditional	1	0.76
VI	Unassessable	3	2.27
Total		**132**	**100.00**

**Table 3:** ADR manifestations of DHI.

Systems/organs	Frequency	Constituent ratio (%)	Manifestations (number of cases)
Skin and its appendages	47	30.13	Pruritus (23), rash (19), sweating (3), and clammy skin (2)
The central and peripheral nervous system damage	34	21.79	Dizziness (17), headache (16), and tics (1)
Extracardiac vascular damage	25	16.03	Superficial phlebitis (12), flushing (9), allergic purpura (1), vasodilation (2), and eye hemorrhage (1)
Systemic damage	12	7.69	Chills (6), high fever (2), fever (1), periorbital edema (1), faintness (1), and chest pain (1)
Heart rate and rhythm disorders	10	6.41	Palpitations (10)
Neurological disorders	10	6.41	Breathlessness (7), anxiousness (3)
Gastrointestinal system damage	9	5.77	Nausea (6), flatulence (2), and vomiting (1)
General damages to the cardiovascular system	4	2.56	Cyanosis (2), hypotension (1), and hypertension (1)
Medication site damage	2	1.28	Local numbness (2)
Respiratory system damage	1	0.64	Dyspnea (1)
Musculoskeletal system damage	1	0.64	Joint disease (1)
Auditory and vestibular dysfunction	1	0.64	Tinnitus (1)
Total	**156**	**100.00**	
